# 恶性血液病患者血流感染的病原菌分布及耐药性单中心回顾性分析

**DOI:** 10.3760/cma.j.issn.0253-2727.2023.06.006

**Published:** 2023-06

**Authors:** 林静 蔡, 小磊 魏, 永强 魏, 绪涛 郭, 雪杰 江, 钰 张, 国攀 余, 敏 戴, 洁瑜 叶, 红升 周, 丹 徐, 芬 黄, 志平 范, 娜 许, 鹏程 史, 丽 宣, 茹 冯, 晓力 刘, 竞 孙, 启发 刘

**Affiliations:** 南方医科大学南方医院血液科，广州 510515 Department of Hematology, Nanfang Hospital, Southern Medical University, GuangZhou 510515, China

**Keywords:** 恶性血液病, 血流感染, 病原菌, 耐药性, Hematological malignancies, Bloodstream infection, Pathogen, Resistance

## Abstract

**目的:**

探讨恶性血液病患者血流感染的发生率、病原菌分布及耐药情况，为临床合理使用抗菌药物提供参考。

**方法:**

回顾性分析2018年1月至2021年12月南方医科大学南方医院血液科收治的恶性血液病合并血流感染患者的临床资料、病原菌分布及耐药性情况。

**结果:**

4年内22 717例次患者共发生582次血流感染，2018–2021年血流感染的发生率依次为2.79％、2.99％、2.79％和2.02％。共分离出599株病原菌，革兰阴性菌487株（81.3％），主要为肺炎克雷伯菌、大肠埃希菌和铜绿假单胞菌；革兰阳性菌81株（13.5％），主要为金黄色葡萄球菌、表皮葡糖球菌和屎肠球菌；真菌31株（5.2％），以热带念珠菌为主。主要肠杆菌科细菌对碳青霉烯类、哌拉西林钠他唑巴坦钠、头孢哌酮钠舒巴坦钠和替加环素的耐药率分别为11.0％、15.3％、15.4％和3.3％。主要非发酵菌对哌拉西林钠他唑巴坦钠、头孢哌酮钠舒巴坦钠和喹诺酮类抗菌药物耐药率分别为29.6％、13.3％和21.7％。81株革兰阳性菌中仅2例对糖肽类抗菌药物耐药。

**结论:**

2018–2021年南方医科大学南方医院血液科恶性血液病患者血流感染以革兰阴性菌为主，不同菌种耐药率差异较大。

## Abstract (en)

**Objective:**

To study the incidence of bloodstream infections, pathogen distribution, and antibiotic resistance profile in patients with hematological malignancies.

**Methods:**

From January 2018 to December 2021, we retrospectively analyzed the clinical characteristics, pathogen distribution, and antibiotic resistance profiles of patients with malignant hematological diseases and bloodstream infections in the Department of Hematology, Nanfang Hospital, Southern Medical University.

**Results:**

A total of 582 incidences of bloodstream infections occurred in 22,717 inpatients. From 2018 to 2021, the incidence rates of bloodstream infections were 2.79％, 2.99％, 2.79％, and 2.02％, respectively. Five hundred ninety-nine types of bacteria were recovered from blood cultures, with 487（81.3％）gram-negative bacteria, such as Klebsiella pneumonia, Escherichia coli, and Pseudomonas aeruginosa. Eighty-one（13.5％）were gram-positive bacteria, primarily Staphylococcus aureus, Staphylococcus epidermidis, and Enterococcus faecium, whereas the remaining 31（5.2％）were fungi. Enterobacteriaceae resistance to carbapenems, piperacillin/tazobactam, cefoperazone sodium/sulbactam, and tigecycline were 11.0％, 15.3％, 15.4％, and 3.3％, with a descending trend year on year. Non-fermenters tolerated piperacillin/tazobactam, cefoperazone sodium/sulbactam, and quinolones at 29.6％, 13.3％, and 21.7％, respectively. However, only two gram-positive bacteria isolates were shown to be resistant to glycopeptide antibiotics.

**Conclusion:**

Bloodstream pathogens in hematological malignancies were broadly dispersed, most of which were gram-negative bacteria. Antibiotic resistance rates vary greatly between species. Our research serves as a valuable resource for the selection of empirical antibiotics.

血流感染（BSI）常见且严重威胁患者生命[1]，我国住院患者BSI的发生率为5.7％，病死率高达28.7％[2]。恶性血液病患者由于自身疾病特点以及化疗、造血干细胞移植、深静脉导管置入、化疗后骨髓抑制等因素[3]–[4]，是发生BSI的高危人群，发生率为11％～38％，病死率高达37.6％[2],[5]–[6]。2020年全国血流感染细菌耐药监测数据显示我国细菌耐药问题日趋严重且地域差异明显[7]，因此明确本地区本病种BSI病原菌分布及耐药情况对指导经验性抗菌药物的选择至关重要。为此我们回顾性分析了2018–2021年南方医科大学南方医院血液科恶性血液病住院患者BSI的病原菌分布及耐药性的情况，以期为临床经验性使用抗菌药物提供参考。

## 病例与方法

1. 病例资料：回顾性分析2018年1月至2021年12月南方医科大学南方医院血液科收治的恶性血液病住院患者中582例次BSI的临床资料。包括性别、年龄、原发病、疾病状态、伴随疾病、其他感染部位、是否发生粒细胞缺乏（粒缺）、7 d及30 d生存情况、病原菌分布和药敏试验结果。

2. BSI诊断标准：BSI是指至少1次血培养中分离细菌或真菌[8]。感染性休克的临床诊断标准为脓毒血症患者经充分容量复苏后仍存在持续性低血压，需缩血管药物维持平均动脉压≥65 mmHg（1 mmHg＝0.133 kPa），且乳酸水平>2 mmol/L[9]。粒缺定义为外周血中性粒细胞绝对计数（ANC）<0.5×10^9^/L。

3. 统计学处理：应用SPSS 20.0软件进行统计学分析。组间定量资料比较采用*t*检验，病原菌分布组间比较采用卡方检验或Fisher确切概率法，双侧*P*<0.05为差异有统计学意义。

## 结果

一、一般情况

2018年1月至2021年12月我院22 717例次恶性血液病住院患者共发生582例次BSI。其中男326例次（56.0％），女256例次（44.0％）。中位年龄为40（28，54）岁，90.5％的患者发生BSI时<60岁。急性髓系白血病（45.9％）、急性淋巴细胞白血病（29.2％）、骨髓增生异常综合征/骨髓增殖性肿瘤（11.2％）是发生BSI最常见的3种恶性血液病。BSI发生时43.5％的患者原发病处于完全缓解状态。70.6％患者为化疗后，25.8％接受造血干细胞移植。

90.2％的患者BSI发生时处于粒缺状态，且12.4％的患者同时合并包括慢性阻塞性肺疾病或慢性支气管炎、糖尿病、其他恶性肿瘤等基础疾病。77.5％的BSI发生时存在侵袭性操作，包括中心静脉置管（PICC）、深静脉置管、胸腔穿刺置管等。44.2％的BSI患者同时合并其他一个或多个部位感染，以肺部感染（30.6％）最为常见，其次为胃肠炎（9.3％）、肛周感染（7.7％）、口腔黏膜炎（6.0％）。

BSI患者出现发热至血培养阳性的平均间隔时间为2.25 d，其中20.8％患者的发热持续时间超过1周，6.9％患者死亡。27.3％的BSI患者发生休克，其中67.2％的休克患者在抗感染、补液及血管活性药物等处理后得以纠正。BSI患者7 d全因死亡率为6.2％，归因死亡率为6.0％；30 d全因死亡率为18.0％，归因死亡率为17.7％。而31例真菌BSI患者 的7 d和30 d的全因死亡率则高达16.1％、41.9％。在40例次发生耐碳青霉烯类肠杆菌（CRE）感染的患者中，50.0％发生感染性休克，7 d全因死亡率为17.5％，30 d全因死亡率为45.0％。

二、BSI的趋势及病原菌分布

既往4年BSI的总发生率为2.56％，2018–2021发生率依次为2.79％、2.99％、2.79％和2.02％。革兰阴性菌BSI 2018–2021年的发生率分别为2.38％、2.56％、2.20％、1.43％，整体呈下降的趋势；其中CRE 2018–2021年在肠杆菌科细菌中的比例分别为16.8％、13.8％、6.8％和8.2％。2018–2021年革兰阳性菌及真菌BSI的发生率分别为0.26％、0.28％、0.46％、0.44％及0.15％、0.12％、0.12％、0.15％，未呈现出下降的趋势。

582例次BSI共分离出的599株病原菌，包括487株（81.3％）革兰阴性菌，81株（13.5％）革兰阳性菌，31株（5.2％）真菌。在革兰阴性菌中，肠杆菌科以肺炎克雷伯菌（185株）、大肠埃希菌（115株）及阴沟肠杆菌（28株）多见，非发酵菌中以铜绿假单胞菌（74株）、鲍曼不动杆菌（22株）及嗜麦芽窄食单胞菌（13株）为主。革兰阳性菌以金黄色葡萄球菌、表皮葡萄球菌以及屎肠球菌为主。真菌以热带念珠菌最为常见。

三、主要革兰阴性菌的耐药情况

检出率最高的三种病原菌是肺炎克雷伯菌、大肠埃希菌以及铜绿假单胞菌，其对碳青霉烯类、头孢哌酮钠舒巴坦钠、阿米卡星的耐药率均低于15％。其他常见的病原菌中，阴沟肠杆菌对替加环素及头孢他啶阿维巴坦的耐药率低于10％，而鲍曼不动杆菌及嗜麦芽窄食单胞菌对绝大多数抗菌药物均存在明显耐药。总体来说，分离出的360株肠杆菌科菌株对碳青霉烯类、哌拉西林钠他唑巴坦钠、头孢哌酮钠舒巴坦钠以及替加环素的耐药率较低，但对喹诺酮类抗菌药物的耐药率较高（表1）。

**表1:** 主要的革兰阴性菌对不同抗菌药物的耐药率（％）

抗菌药物	肠杆菌				非发酵菌		
	肺炎克雷伯菌（185株）	大肠埃希菌（115株）	阴沟肠杆菌（28株）		铜绿假单胞菌（74株）	鲍曼不动杆菌（22株）	嗜麦芽窄食单胞菌（13株）
氨苄西林	100.0	88.3	100.0		100.0	100.0	100.0
哌拉西林	39.8	80.5	52.6		3.7	60.0	100.0
头孢他啶	17.8	22.2	46.4		5.6	60.0	16.7
头孢噻肟	25.6	48.3	38.9		98.2	60.0	100.0
头孢唑林	95.9	100.0	94.7		100.0	100.0	100.0
头孢吡啉	66.7	100.0	/		/	/	/
头孢吡肟	24.2	38.9	46.4		5.6	66.7	/
氨曲南	20.4	32.5	46.4		9.1	100.0	100.0
阿米卡星	9.8	6.1	39.3		2.7	60.0	100.0
庆大霉素	30.1	46.6	26.3		9.1	57.1	100.0
环丙沙星	30.8	53.2	53.6		7.4	83.3	100.0
莫西沙星	17.2	48.9	21.1		0.0	60.0	/
复方磺胺甲唑	59.9	85.0	71.4		96.7	81.3	18.2
四环素	62.9	87.2	31.6		98.3	55.6	83.3
阿莫西林克拉维酸	15.2	14.1	94.4		98.2	100.0	100.0
氨苄西林舒巴坦	32.1	28.6	100.0		98.2	66.7	100.0
哌拉西林钠他唑巴坦钠	16.0	12.1	33.3		10.4	85.7	100.0
亚胺培南	10.4	7.1	42.9		11.1	88.9	100.0
美罗培南	10.4	7.9	40.7		8.3	81.0	100.0
氯霉素	50.4	50.5	47.4		89.7	77.8	33.3
左氧氟沙星	18.2	51.0	38.5		11.8	70.6	25.0
替加环素	8.0	15.2	7.1		70.6	27.8	0.0
头孢哌酮钠舒巴坦钠	12.4	9.3	43.5		7.1	42.1	/
头孢他啶阿维巴坦	16.7	28.6	0.0		0.0	75.0	0.0

注 /：未进行药敏试验

四、主要革兰阳性菌的耐药情况

金黄色葡萄球菌对青霉素类抗菌药物几乎全部耐药，表皮葡萄球菌对青霉素类、喹诺酮类以及氨基糖苷类抗菌药物有较高的耐药率，几乎均在50％以上，但未检测到对万古霉素、替考拉宁及利奈唑胺耐药的金黄色葡萄球菌及表皮葡萄球菌。屎肠球菌对绝大多数抗菌药物存在明显耐药，对替考拉宁、万古霉素的耐药率亦高达11.1％，但未检测到对利奈唑胺耐药的菌株（表2）。

**表2:** 主要的革兰阳性菌对不同抗生素的耐药率（％）

抗菌药物	金黄色葡萄球菌（17株）	表皮葡萄球菌（16株）	屎肠球菌（9株）
氨苄西林	100.0	100.0	100.0
头孢西丁	100.0	/	100.0
阿米卡星	0.0	0.0	100.0
庆大霉素	6.3	62.5	100.0
妥布霉素	0.0	0.0	100.0
环丙沙星	6.7	83.3	100.0
复方磺胺甲唑	6.7	81.3	100.0
甲氧氨苄嘧啶	0.0	/	100.0
克林霉素	10.0	54.5	100.0
红霉素	40.0	68.8	100.0
呋喃奎因	0.0	0.0	77.8
替考拉宁	0.0	0.0	11.1
万古霉素	0.0	0.0	11.1
奎奴普丁达富普丁	7.1	/	0.0
四环素	42.9	18.2	77.8
呋西地酸	/	/	100.0
利福平	13.3	31.3	88.9
阿莫西林	0.0	100.0	/
青霉素	100.0	100.0	/
左氧氟沙星	0.0	50.0	/
莫西沙星	0.0	33.3	/
氧氟沙星	/	50.0	/
替利霉素	/	0.0	/
利奈唑胺	0.0	0.0	0.0
厄他培南	/	/	/
美罗培南	/	/	/
氯霉素	/	/	/

注 /：未进行药敏试验

## 讨论

目前全球BSI的发生率及死亡率呈持续增长的趋势[10]，在我国已成为继下呼吸道感染和尿路感染的第三大院内感染[11]。恶性血液病患者由于疾病本身和治疗相关的免疫抑制更易发生BSI且死亡率更高[5],[12]。本研究中我们回顾性分析了2018–2021年南方医科大学南方医院血液科恶性血液病住院患者BSI的发生率，结果显示既往4年BSI的发生率为2.56％，总体呈逐渐下降趋势，但是7 d和30 d的全因死亡率为6.2％和18.0％。因此明确本地区的病原学分布、耐药性以及临床特征，为经验性抗菌药物的选择提供依据至关重要。

本研究纳入582例次BSI，主要发生于急性髓系白血病、急性淋巴细胞白血病和骨髓增生异常综合征/骨髓增殖性肿瘤为主，治疗方式以化疗和造血干细胞移植患者。90.2％的BSI患者处于粒缺状态。44.2％的BSI患者同时合并其他部位感染，以肺部感染（30.6％）最为多见，其次为胃肠炎、肛周感染、口腔黏膜炎，与张国扬等[13]的报道一致。但是否为原发感染灶难以明确。

本组病例中分离出599株病原菌，以革兰阴性菌（81.3％）为主，检出率最高的3种病原菌分别是肺炎克雷伯菌、大肠埃希菌和铜绿假单胞菌。肺炎克雷伯菌总体耐药率低于10.5％。大肠埃希菌对青霉素类、喹诺酮类抗菌药物的耐药率接近50.0％，而对头孢菌素、碳青霉烯类和阿米卡星的耐药率低于10.0％。肠杆菌科细菌（360株）占革兰阴性菌的73.9％，总体对碳青霉烯类抗菌药物、哌拉西林钠他唑巴坦钠、头孢哌酮钠舒巴坦钠以及替加环素的耐药率均低于20.0％，但对喹诺酮类抗菌药物的耐药率较高。引起高死亡率的CRE 2018-2021年在肠杆菌科细菌中的比例分别为16.8％、13.8％、6.8％和8.2％，呈逐年下降趋势。原因可能和科室经验性抗菌药物的选择有关。自2019年起根据科室每周耐药菌的特点，每3～4个月调整科室经验性抗菌药物选择方案，后续根据药敏结果靶向选择。

革兰阳性菌中检出率最高的分别是金黄色葡萄球菌、表皮葡萄球菌以及屎肠球菌。本研究未检出对万古霉素、替考拉宁以及利奈唑胺耐药的金黄色葡萄球菌和表皮葡萄球菌，而屎肠球菌对绝大多数抗菌药物存在明显耐药，对万古霉素的耐药率为11.1％，高于国内其他研究[14]–[15]，可能与本组病例屎肠球菌总体检出率（9株）低相关，也可能是本单位病原菌本身的特点。本组病例中共分离出31株真菌，占所有BSI的5.5％，但是30 d内的死亡率高达41.9％。

综上所述，本研究所纳入的恶性血液病患者在发生BSI时多处在粒缺期，易伴随其他部位的感染，其中以肺部感染最为常见。病原菌分布以革兰阴性菌为主，以肺炎克雷伯菌、大肠埃希菌以及铜绿假单胞菌最为常见。肠杆菌科、非发酵菌、革兰阳性菌和真菌对抗菌药物表现出不同规律，耐药性复杂多样。
